# In situ observation of helium and argon release during fluid-pressure-triggered rock deformation

**DOI:** 10.1038/s41598-020-63458-x

**Published:** 2020-04-24

**Authors:** Clément Roques, Ulrich W. Weber, Bernard Brixel, Hannes Krietsch, Nathan Dutler, Matthias S. Brennwald, Linus Villiger, Joseph Doetsch, Mohammadreza Jalali, Valentin Gischig, Florian Amann, Benoît Valley, Maria Klepikova, Rolf Kipfer

**Affiliations:** 1 ETH Zürich, Department of Earth Sciences, Sonneggstrasse 5, 8092 Zürich, Switzerland; 2 University Rennes 1, Géosciences Rennes, UMR 6118, Av. du Général Leclerc, 35042 Rennes, France; 3 Eawag - Swiss Federal Institute for Aquatic Science and Technology, Department of Water Resources and Drinking Water, Ueberlandstrasse 133, 8600 Dübendorf, Switzerland; 4 University of Oslo, Department of Geosciences, Sem Sælands vei 1, 0371 Oslo, Norway; 5 University of Neuchâtel, Center for Hydrogeology and Geothermics, Rue Emile-Argand 11, 2000 Neuchâtel, Switzerland; 6 RWTH Aachen, Department of Engineering Geology and Hydrogeology, Lochnerstrasse 4-20, 52064 Aachen, Germany; 7 CSD INGENIEURE AG, Hessstrasse 27D, 3097 Liebefeld, Switzerland; 8 University of Lausanne, Applied and Environmental Geophysics group, Institute of Earth Sciences, Lausanne, 1015 Switzerland; 9 ETH Zürich, Department of Environmental System Science, Universtaetstrasse 16, 8092 Zürich, Switzerland; 10 ETH Zürich, Department of Earth Sciences, Institute of Geochemistry and Petrology, Sonneggstrasse 5, 8092 Zürich, Switzerland

**Keywords:** Geochemistry, Hydrogeology, Seismology

## Abstract

Temporal changes in groundwater chemistry can reveal information about the evolution of flow path connectivity during crustal deformation. Here, we report transient helium and argon concentration anomalies monitored during a series of hydraulic reservoir stimulation experiments measured with an *in situ* gas equilibrium membrane inlet mass spectrometer. Geodetic and seismic analyses revealed that the applied stimulation treatments led to the formation of new fractures (hydraulic fracturing) and the reactivation of natural fractures (hydraulic shearing), both of which remobilized (He, Ar)-enriched fluids trapped in the rock mass. Our results demonstrate that integrating geochemical information with geodetic and seismic data provides critical insights to understanding dynamic changes in fracture network connectivity during reservoir stimulation. The results of this study also shed light on the linkages between fluid migration, rock deformation and seismicity at the decameter scale.

## Introduction

Changes in the crustal stress state caused by natural or human-induced subsurface fluid overpressures can lead to brittle rock mass damage, from the formation of grain-scale microcracks to the failure of kilometric-scale faults^1^. Characterizing the timing and spatial extent of crustal deformation is critical for both industrial applications, including geothermal, oil and gas production, and improving our mechanistic understanding of earthquakes. From this perspective, seismic and geodetic monitoring systems generally provide the vast majority of data collected. Several authors have also proposed that geochemical anomalies may be used as proxies for rock deformation^2–5^.

Fluids in the Earth's crust display highly variable geochemical traits due to the spatial and temporal variability in fluid recharge composition, fluid-rock interactions and residence times. Previous studies have shown extremely variable distributions of residence times, ranging from decades^6^ to several hundred or even millions of years for fluids stored in low-permeability rocks^7,8^. In this context, the fluid composition may evolve from diluted waters with modern signatures in recharge areas to saline fluids in the deeper crust, where the fluid composition tends to equilibrate with the host rock mineralogy through dissolution/precipitation processes. By analyzing specific dissolved chemical tracers, one can reconstruct the origin of fluids and gain insights into their recharge, percolation and storage conditions^9,10^.

From this perspective, noble gases such as radon (Rn), helium (He) and argon (Ar) have received particular attention in recent decades because of their widespread occurrence in the subsurface and their low chemical reactivity, proving to be ideal tracers to track the origin of fluids, analyze fluid-rock interactions and determine residence times^7,8,11–14^. Concentrations of dissolved noble gases in subsurface fluids and their isotopic compositions are driven by two main mechanisms: 1) water/air partitioning during recharge, which is sensitive to atmospheric pressure, temperature and salinity, and 2) radiogenic production in the crust through radioactive alpha-decay of heavy elements (mainly uranium, thorium or potassium) combined with the accumulation of enriched fluids from deeper reservoirs (e.g., the upper mantle)^9^.

Chemical anomalies created by earthquakes have been explained by the mixing of fluids from different sources (with different chemical compositions) inhibited by changes in flow pathway geometry and hydraulic properties during stress build-up and failure^15,16^. Laboratory studies have also observed the emergence of concentration anomalies in noble gases during rock deformation experiments^17–20^. The authors argued that the progressive increase in microcrack surfaces during increasing stress allows the remobilization of accumulated radiogenic gases and even prior macroscopic failure. While those previous studies suggested that changes in dissolved noble gas concentration could be used as a proxy to track *in situ* stress modifications, field evidence and a description of the mechanisms responsible for fluid remobilization and mixing at the scale of discrete fracture systems are still missing to our knowledge. Our aim in this study is to bridge this knowledge gap through a joint analysis of hydraulic, seismic, deformation and geochemical data gathered during an *in situ* stimulation experiment.

## Experiment description

**Figure 1:**
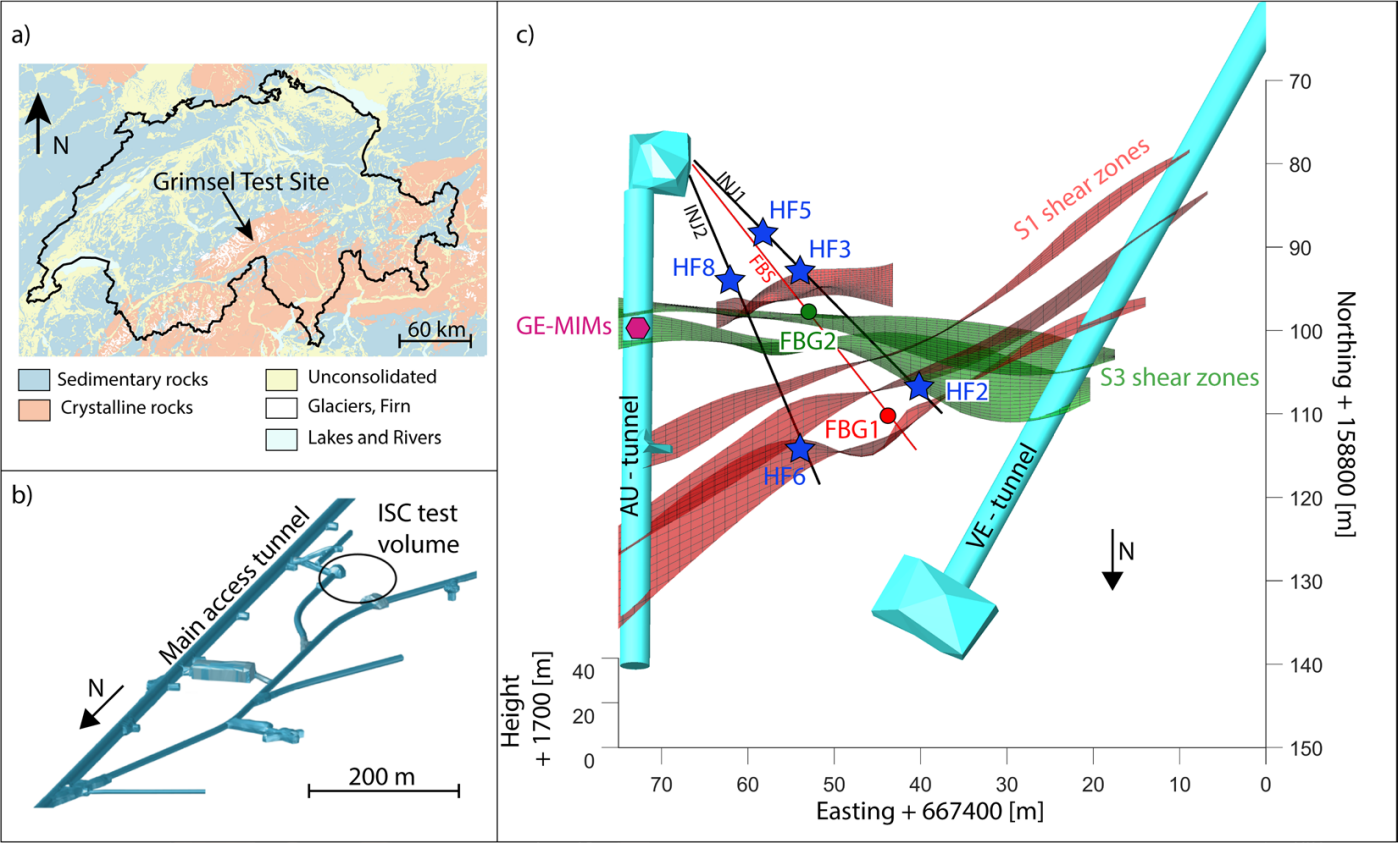
Overview and location of the experimental test volume for the *In situ* Stimulation and Circulation (ISC) project: (**a**) geological map of Switzerland (modified from the website https://map.geo. admin.ch/, Federal Office of Topography), (**b**) tunnel network and location of the investigated volume, (**c**) geological model displaying the main shear zones (modified after Krietsch *et al*., 2018^23^), along with the main boreholes used for injection (INJ1 and INJ2) and monitoring (FBS), the locations of stimulation (HF#) and strain monitoring (Fiber-Bragg-Grating, FBG) intervals, and the location of the *in situ* gas equilibrium membrane inlet mass spectrometer (GE-MIMS[24]) used for monitoring dissolved noble gas concentrations. For a more complete description of the geology of the investigated volume and the monitoring system deployed, readers are referred to Krietsch *et al*., 2018 and Amann *et al*., 2018^22,23^.

The study was carried out as part of a series of six controlled hydraulic fracturing (HF) experiments^21^ that took place in May 2017 at the Grimsel Test Site, Switzerland (*In situ* Stimulation and Circulation project^22^, www.grimsel.com, Fig. 1). The site consists of a network of tunnels, operated by the Swiss National Cooperative for the Disposal of Radioactive Waste (NAGRA), and these tunnels are located up to 480 m below ground surface in the central Aar granite and granodiorite formations^23^. The stimulated rock volume comprises a relatively intact granodiorite intersected by six subvertical shear zones that can be grouped into 2 sets: ductile shear zones S1 (red in Fig. 1c) and brittle-ductile shear zones S3 (green in Fig. 1c), which form the main transmissive fractured zones^23^.

In this study, we refer to a ‘fracture’ as a single discontinuity that can be identified on optical or acoustic logs (resolution of ~1 mm). A ‘shear zone’ is described as a zone characterized by strong strain concentration that may contain several fractures visible in the optical or acoustic logs. Finally, ‘intact rock’ is understood to be a section of the rock mass with the absence of fractures detectable from the optical and acoustic logs.

From May 16^th^ to 19^th^, six high-pressure fluid injections (resulting pressure below 10 MPa during the fracture propagation phase) were performed in two injection boreholes (INJ1-2 in Fig. 1c, labeled HF#) at different depth intervals by isolating 1 m intact rock with double packer systems. A dense network of boreholes was equipped with monitoring systems aimed at capturing hydraulic, strain and seismic responses during stimulation (see Methods). As a complement, a portable gas equilibrium membrane inlet mass spectrometer (GE-MIMS^24^) was installed at a tunnel inflow, where groundwater naturally seeps from an extensional fracture located between the two targeted S3 shear zones. The GE-MIMS allowed monitoring of near-real-time He, N_2_, and Ar concentrations dissolved in the pore water with a sampling interval of 8 minutes (see Methods). We anticipated potential effects of the low flowrates involved at the seeping fracture that could lead to incomplete gas/water equilibration at the extraction membrane of the GE-MIMS, by normalizing the He and Ar concentrations by the concentrations of dissolved N_2_ (only of atmospheric origin in the case of this experiment). This ensures that the observed He/N_2_ and Ar/N_2_ ratios only reflect the variations of dissolved He and Ar in the fluid (see Methods).

## Results

### Strain, seismic and hydraulic responses induced by high-pressure fluid injection

**Figure 2:**
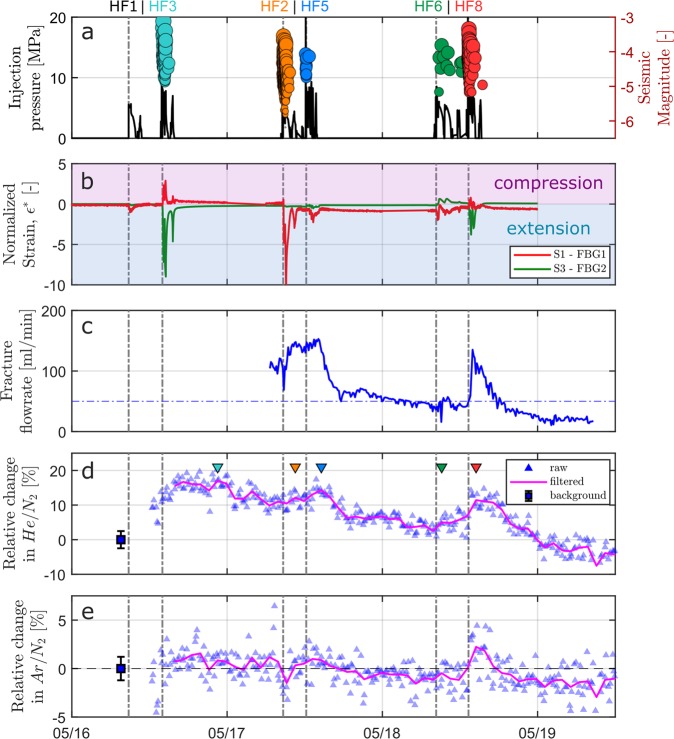
Temporal evolution of (**a**) injection pressure drawn as a continuous black line together with the magnitude of seismic events, which are represented by circle symbols. The diameter of the circles are scaled with the magnitude and the colors differentiate the responses from the different HF experiments; (**b**) normalized strain ${\epsilon }^{\ast }=\epsilon /{\boldsymbol{std}}(\epsilon )$ [-] for two selected strain sensors located in both S1 and S3 shear zones. Strain has been normalized by its standard deviation to better appreciate the shapes of both time series. Positive values indicate compressional regimes, while negative values indicate extensional regimes; (**c**) fracture discharge rates monitored at the GE-MIMS station. The horizontal dashed line represents the average discharge rate prior to stimulation; (**d**) relative changes in He/N_2_ from background [%] and (**e**) relative changes in Ar/N_2_ from background [%]. The background average ratio in He/N_2_ and Ar/N_2_ are shown as blue squares. Background error bars are 2σ and represent the typical analytical error. Time series of raw data are shown as blue triangles, and low-pass filtered ratios are represented by magenta lines. The different stimulation experiments are labeled with HF# at the top of the figure. Colored triangles in subpanel c identify the peaks in He/N_2_ concentrations following each HF# experiment, which are further analyzed in Fig. 3.

The evolution of strain monitored in the S1 and S3 shear zones during the different high-pressure injections showed that both extension and compression deformation were involved. (Figure 2b, positive values for the compressional regime and negative values for the extensional regime). Deformation patterns show variability across the different HF experiments with amplitudes ranging from −386.3 µε extension to +28.5 µε compression (Table 1SM in Supplementary Material). As a general interpretation of the main processes involved, we argue that while the extensional regime is dominant at the locus of injection due to local increasing pore pressure, the nearby structures experience a compressive response to balance the stress perturbation. As the injection progresses, the stress perturbation diffuses away from the stimulation interval, enabling fracture initiation and reactivation. After a peak in deformation is monitored almost simultaneously with the maximum injection pressure, the strain decreases back to values close to the initial background. This recovery is mostly controlled by the combined effect of fluid pressure diffusion within the fracture network and elastic relaxation of the rock. Although the strain signal appears to be mainly dominated by reversible processes, the residual strain observed in the time series indicates that irreversible deformation due to imperfect normal fracture closing is involved^21^.

A total of 774 seismic events were located during the whole experiment, with magnitudes ranging from −6.2 to −3.1 (Fig. 2a). Seismic events strongly correlate with injection activities, and even though similar fluid volumes were injected during all experiments following similar injection protocols, strong variability in seismic characteristics was observed across a single HF experiment (Table 1SM in Supplementary Material) - *i.e*., located seismic events, maximum induced magnitudes, seismically activated areas (determined with planar seismic cluster geometries) and Gutenberg-Richter *b*-values, as well as seismogenic indices, ∑ (determined for experiments exhibiting more than 50 induced events). The Gutenberg-Richter *b*-values and the seismogenic indices, ∑, are of particular interest when comparing the intensity of seismic responses across different tests^25^. Note that a low b-value (*i.e*., comparably larger magnitude events with respect to small magnitude events) and a high seismogenic index (i.e., comparably increased seismogenic reaction to a unit volume of fluid) represents a comparable, more intensive seismic response^26^ (Fig. 1SM in Supplementary Material).

High-pressure injection activities promoted increases in discharge rates monitored at the GE-MIMS station during HF2, 5, 6 and 8 (Fig. 2c). These fast reactions at the tunnel outlet reveal efficient connectivity and high hydraulic diffusivities (on the order of 0.1–1 m/s) of the stimulated fracture network.

The main differences in seismic, deformation and hydraulic responses among the HF experiments allow for the definition of two main clusters, which are primarily constrained by their location with respect to the S3 shear zones: **(I) HF3, 5 and 8** were all carried out south of the S3 shear zones. All of these experiments showed high injection pressures, fast pressure decay after injections and relatively low fluid recovery. This finding suggests that the stimulated fractures progressively connected to the preexisting transmissive fracture system, allowing the injected fluid to propagate efficiently within the network^21^. HF3 and HF8 induced 70 and 183 micro-seismic events, respectively, for which deformation patterns were mainly dominated by the extensional regime along the S3 shear zones. While HF3 showed a dispersed seismic cloud over a large rock volume, seismic events located during HF8 were organized along a well-identified fracture plane (estimated area of 235 m^2^, see Table 1SM in Supplementary Material) propagating toward the S3 shear zone. The seismic response of experiment HF3 (b = 1.55, ∑: −4.8) increased compared to experiment HF8 (b = 2.66, ∑: −9.0), and the largest magnitude event of the experiment series was monitored during HF3. HF5 showed the lowest number of seismic events (11), with a smaller seismically activated area (<10 m^2^) and a compressional regime along S3^21,26^. **(II) HF2 and 6** were performed within the S1 shear zones – *i.e*., north of the S3 shear zones. Pressure diffused at a slower rate within the reservoir, and fluid recovery was higher after the shut-in phase, suggesting lower connectivity to the preexisting transmissive network. Extensional deformation along S1 was dominant during HF2, while HF6 was mainly linked to compressional deformation of S1 and the extension of S3^21^. HF6 did not induce a significant number of microseismic events, which was most likely because the experiment was conducted in an interval that contained preexisting S1 structures that were hydraulically stimulated in a previous experiment series^26^. HF2 promoted the highest number of induced seismic events, an activated surface area of approximately 95 m^2^ and the largest seismic response of the HF experiment series^26^ (b = 1.35, ∑: −4.0, also see Table 1SM and Fig. 1SM in Supplementary Material).

### Geochemical tracing of involved fluid origins before stimulation

Pore water sampled at the monitoring station shows low mineralization with an electrical conductivity of 300 µS/cm, a pH of 9 and a temperature of 12 °C (Table 2SM in Supplementary Material). N_2_ and Ar concentrations of 1.4^.^10^−2^ ccSTP/g and 3.7^.^10^−4^ ccSTP/g, respectively, indicate that both elements are oversaturated by approximately 25% with respect to atmospheric-equilibrated water. This excess of N_2_ and Ar is typically assigned to an “excess-air” phenomenon, which implies oversaturation in gas species due to the partial dissolution of entrapped air bubbles during recharge^27,28^. Ar shows 2.5% more oversaturation than N_2,_ while He is also significantly enriched with respect to atmospheric-equilibrated water of approximately 800% (3.4^.^10^−7^ ccSTP/g, Table 2SM in Supplementary Material). This oversaturation in He, combined with the slight enrichment in Ar over N_2_ of ~2.5%, shows the accumulation of radiogenic-produced noble gases, which is confirmed by a low ^3^He/^4^He ratio of the excess He (taking a value of 2.2^.^10^−7^, which is 6.1 times lower than the atmospheric ratio^29^ of 1.384^.^10^−6^; please refer to Table 2SM in the Supplementary Material and Methods).

**Figure 3:**
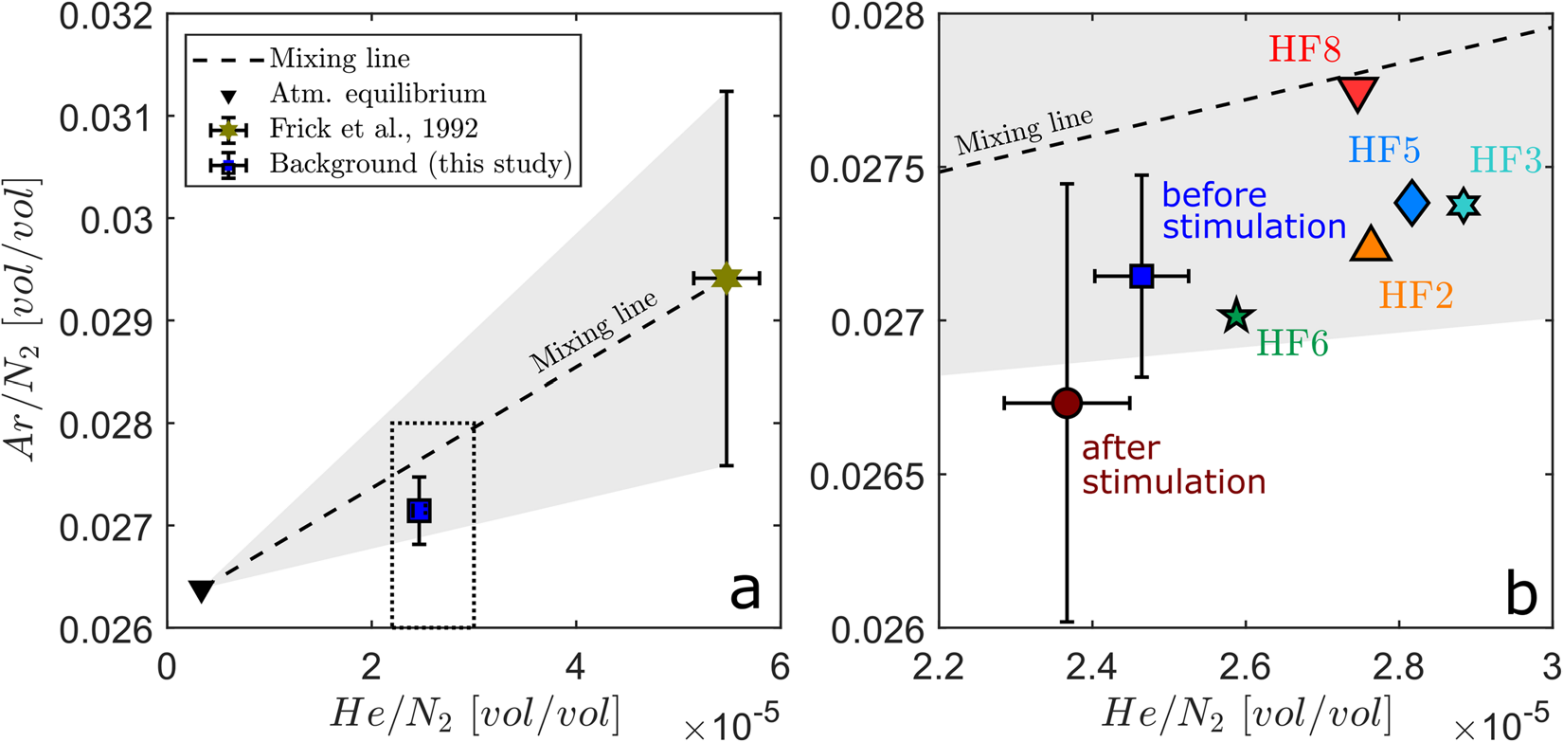
(**a**) Ar/N_2_ as a function of He/N_2_. The mixing line (short dashed line) from atmospheric (black reversed triangle) to (He, Ar)-enriched fluid end-members (olive star) was collected in 1982 during the realization of exploration boreholes and reported in Frick *et al*.^30,31^. The confidence area for binary mixing is drawn as a gray triangle accounting for the variability in the Ar and N_2_ concentrations measured in the samples collected in 1982. The background average ratio and standard deviations are shown as blue squares and error bars. (**b**) is a zoom of the concentration range of interest (corresponding to the dashed rectangle in panel a) with peak concentrations presented with different symbols and colors for the different HF# experiments (refer to Fig. 2d for the locations of peak concentrations identified in the time series of He/N_2_). Concentrations measured after the stimulation experiment are shown as a wine-colored circle. Error bars are 2σ.

Even larger excesses in He, Ar and N_2_ (*i.e*., concentrations exceeding the expected atmospheric equilibrium concentrations) were also determined in pore waters collected during early sampling campaigns on exploration boreholes in 1982^30,31^ (Fig. 3a and Table 2SM in Supplementary Material). These differences indicate that the initial pore water sampled in 1982 could have slightly different meteoric origins with higher soil temperatures at recharge, different residence times controlled by local geological heterogeneity, or that the present-day concentrations may be diluted by modern fluids injected during recent activities performed in the tunnel, *i.e*., extensive drilling, hydraulic testing, and *in situ* experiments. Nevertheless, the relationship between He/N_2_ and Ar/N_2_ (Fig. 3a) is remarkably aligned along a mixing line between the non-atmospheric fluid enriched in He and Ar that was collected in 1982 and modern air-equilibrated meteoric water. Note that the background concentration (Fig. 3a) plots in the mixing triangle (in gray on Fig. 3) defined by the variability in Ar/N_2_ ratios measured in the pore water sampled in 1982. This confirms that binary mixing between the two geochemical end-members explains the fluid composition seeping from the fracture where the GE-MIMS was installed. It also allows us to estimate that the (He, Ar)-enriched end-member contributes approximately 25% to the background fluid composition.

### Geochemical anomalies involved during reservoir stimulation

The first two injections, HF1 and HF3, performed on May 16^th^ (Fig. 2) caused a significant increase in He/N_2_ ratios of up to 17% relative to the background values, while Ar/N_2_ ratios remained close to background values (Fig. 2d,e). After this sudden increase, the He/N_2_ ratios tend to continuously decrease. This trend was interrupted by enhanced ratios in response to the following stimulation (HF2, 5, 6 and 8). Experiments HF3 and 8, which were carried out south of the transmissive shear zones and the closest to the GE-MIMS monitoring station (24 and 17.4 m geometrical distance from locus of injection, respectively, Table 1SM in Supplementary Material), were associated with an abrupt increase in the He/N_2_ anomalies. HF5 fostered a much smaller anomaly, while HF2 and HF6 were hardly detectable against the given experimental uncertainty of the dissolved gas analysis. In response to HF8, an ~2% increase in the Ar/N_2_ ratio was observed.

Enhanced He/N_2_ ratios correlate with the occurrence of microseismic events (Fig. 2a) and increase in discharge rates (Fig. 2c), indicating that He-enriched fluids are remobilized within the fracture network during the injection cycles. The majority of normalized peak concentrations lines up subparallel to a binary mixing line of the two respective end-members and within the confidence area (Fig. 3b), suggesting that no other type of fluid is involved. Mixing fractions of the He/N_2-_ and Ar/N_2_-enriched fluid ranged from 20 to 45%, whereby maximum values were linked to stimulation experiments HF3 (33%), HF5 (33%) and HF8 (45%), while HF2 and HF6 showed lower values of 28% and 21%, respectively. It is remarkable that these two sets of experiments identified based on the differences in geochemical anomalies match the two clusters that have been previously identified as having similar hydromechanical responses. Experiments HF3, 5 and 8, which were performed south of the S3 shear zone, have enabled the development of new fractures that effectively connected the preexisting transmissive fracture network and were followed by higher He/N_2_ anomalies. However, experiments HF2 and HF6, which were undertaken north of the S3 shear zone, induced poor fracture connection with the transmissive S3 shear zone and were followed by lower He/N_2_ anomalies.

The day after the last experiment, He/N_2_ and Ar/N_2_ reached a lower level than before the experiments, with a contribution of approximately 11.5% from the (He, Ar)-enriched fluid. Figure 3b also shows that the concentrations plot outside the confidence area for binary mixing. This difference may result from the modifications in the fracture network connectivity due to the stimulation, which tends to increase the contribution of a different source of water that was not previously identified (*i.e*., meteoric water recharged under different temperature and pressure conditions).

## Discussion

### He and Ar concentration anomalies provide insights into new flow pathways formed in response to reservoir stimulation

Our assessment demonstrates the ability of GE-MIMS technology to monitor fluid remobilization in response to *in situ* rock deformation. The temporal scales of the processes involved not only require a high-frequency monitoring system^24,32^ but also a multi-tracer approach, *i.e*., He, Ar and N_2_ in the case of the GTS. Complementing the findings of previous experiments performed in the laboratory^17–20^, as well as those advanced from large-scale observations in the context of earthquakes^5^, we interpret these anomalies as the release of radiogenic He (and Ar) stored in the rock mass that could be transported within the newly created and pre-existing fracture system. While the identification of the source of the mobilized fluids is still speculative, two complementing hypotheses on its origin are proposed: (1) radiogenic He (and Ar) accumulated in the rock matrix is released by the formation of new fracture areas; and/or (2) the shearing of pre-existing fractures induces the remobilization of stagnant fluids, with higher residence times enriched in radiogenic He and Ar, trapped in lower transmissivity zones of the fracture network. In both scenarios, the improved connectivity resulting from the stimulation, combined with the high pore pressure gradients involved, allows the transport and dispersion of those fluids with their respective noble gas signature away from their original location.

The magnitude and temporal scaling of the produced concentration anomalies depend not only on concentration contrasts between the involved fluids (geochemical end members) but also on flow connectivity and transport properties of the fracture system through which the fluids move. The time lag between the high-pressure injection and the peak in noble gas concentrations may provide estimates of the average fluid velocities involved if the original location of the activated fluids is known, *i.e*., assuming that most of the (He, Ar)-enriched fluid originates from the vicinity of the injection point where the fractures are created or reactivated. In the case of this experiment, advective velocities of 10^−3^ to 10^−2^ m/s can explain the observed time lags. The rate at which concentration anomalies dissipate within the fracture network may provide information on how much the fluid is dispersed within the reservoir, with the dispersion length scaling with the square root of the residence time^33^. We observed a He/N_2_ restitution rate during HF3 that suggested higher dispersion than for the other experiments, with an anomaly that persisted during the whole week of the experiment. HF3 also showed a dispersed seismic response, suggesting the activation of multiple fractures in the surrounding volume, enhancing the dispersion of (He, Ar)-enriched fluids. In contrast, the abrupt and sharp He/N_2_ anomaly linked to HF8 relaxes to the corresponding background values within 10 hrs. Since HF8 was performed closest to the GE-MIMs and induced the creation of a single fracture plane that rapidly connected to the transmissive S3 shear zone^21,26^, the dispersion of (He, Ar)-enriched fluids is expected to be lower than in the case of HF3.

## Conclusion

In this paper, we provide *in situ* evidence of He and Ar anomalies occurring during the deformation of a crystalline fractured system at the decameter scale. We observed clear differences in the magnitude and temporal dynamics of these anomalies for different clusters of stimulation experiments that displayed differences in their hydro-seismo-mechanical responses. Complementing previous hypotheses drawn from laboratory experiments, we speculate that the remobilization of (He, Ar)-enriched fluids is principally controlled by (1) the extent of the deformed rock mass and the area of the ruptured fractures, which may control the amount of (He, Ar)-enriched fluids released; (2) the type of deformation, which may lead to different concentration anomalies. The generation of a single fracture plane in intact rocks leads to a stronger (He, Ar) signal, typically experiment HF8, than in the case of the reactivation of multiple fractures that may disperse the signal, typically experiment HF3; (3) the efficiency of the newly generated fracture of connecting with the preexisting transmissive fracture system, allowing its transport within the reservoir; and (4) the distance of the source of (He, Ar)-enriched fluids from the monitoring point.

Overall, high-frequency noble gas measurements in fractured rocks may help to improve the conceptualization of the nature and evolution of flow paths in response to stimulation operations, and complement the information obtained from hydraulic, deformation and seismic monitoring systems. Specifically, the analysis of the magnitude and temporal scaling of hydrogeochemical changes, such as the described noble gas anomalies, may inform about the evolution of the hydraulic connectivity and the transport properties of the stimulated fracture network, which is information that is not accessible through classic seismic and geodetic methods.

From similar perspectives, valuable information could be gained through high-frequency noble gas monitoring in both CO_2_ injection and oil and gas extraction projects, where undesired gas migration pathways toward shallower aquifers could be identified during operation^34,35^. Monitoring noble gas concentrations may also help understand the impact of natural seismic events on regional aquifer connectivity, providing critical information to link seismicity to fluid migration in the Earth's crust.

## Methods

### Injection protocol

Six high-pressure fluid injections were performed in two injection boreholes (INJ1-2 in Fig. 1, labeled HF#) by isolating 1 m of intact rock with double packer systems. The targeted injection intervals had a constant base length of 1 m. Prior to each HF test, the interval integrity and proper packer sealing were tested with a short pulse test. HF tests started with a flow rate-controlled (5 l/min) injection cycle that lasted for 30 seconds to initiate fracturing (i.e., formation breakdown), followed by shut-in and bleed-off phases. Subsequently, the fracture was propagated with flow rate-controlled injections (up to 100 l/min). A total of 1,000 liters of fluid was injected during each test over all cycles. Each injection cycle was followed by a shut-in phase. During HF5, HF6 and HF8, water and a rheology modifier (mixture with xanthan gum polymer) were used to achieve a viscosity-dominated propagation regime^21^. The latter consists of a xanthan-salt-water mixture providing 35 times higher viscosity than water. For the tests undertaken with xanthan-salt-water, a supplementary flushing cycle was performed to flush as much xanthan as possible out of the fracture. The last cycle was a pressure-controlled step test to evaluate the post-stimulation injectivity of the created hydraulic fracture and to estimate the stress acting normal to the hydraulic fracture (jacking pressure). Note that HF1 is not considered in this study because it suffered from logistical issues that prevent any reliable monitoring and interpretation.

### Deformation monitoring

To obtain quantitative information about the deformation field inside the test volume, three boreholes (referred to as FBS boreholes in Fig. 1) were drilled, and each borehole was equipped with 20 Fiber-Bragg-Grating strain sensors. The sensors provide one-dimensional borehole-parallel information about local deformations. Prior to installation, fractures were mapped along the borehole from optical and acoustic televiewer logs^36^. Care was taken to ensure that the strain sensors covered the different geological features of interest, i.e., including preexisting fractures, shear zones and intact rock sections. The sensors have a length of 1 m, accuracy of 1 microstrain, and resolution of 0.1 microstrain and were measured at a sampling rate of 1000 Hz. The strain signals described in this paper (from borehole FBS1 at 22.35 m and 42.2 m depths) each cover one of the main shear zones (S3.1 and S1.3) targeted by the stimulations, providing information about the extent of deformation and its temporal variability.

### Seismic monitoring

Seismicity was monitored during the injection^26^, shut-in and bleed-off phases of the six HF experiments using 26 highly sensitive piezoelectric acoustic emission sensors. Eight of the acoustic emission sensors were deployed in four monitoring boreholes in proximity to the injection intervals. Seismicity was recorded continuously at a sampling rate of 200 kHz. Seismic event detection was performed using a standard recursive short-time-average/long-time-average coincidence trigger, whereby picking was carried out manually. Seismic event location was performed in an anisotropic, homogeneous velocity model. An amplitude-based magnitude M_A_ was estimated for located seismic events and corrected for angle-dependent sensitivity variations and coupling quality of acoustic emission sensors. The amplitude-based magnitudes M_A_ were finally adjusted to absolute magnitudes M_W_ determined for a subset of larger magnitude seismic events. The absolute magnitudes M_W_ were estimated from acoustic emission sensors installed in nearby tunnels that were collocated with calibrated accelerometers. The collocation of acoustic emission sensors and accelerometers allowed for a cross-calibration of the acoustic emission sensors. Finally, to determine absolute magnitudes M_W_, the theoretical displacement source spectrum introduced by Boatwright (1978)^37^ was fitted to the recorded displacement spectrum on the cross calibrated acoustic emission sensors^26^.

### Continuous noble gas monitoring

A portable gas equilibrium membrane inlet mass spectrometer (GE-MIMS, Gasometrix GmbH) was installed at a nearby natural seeping zone related to the transmissive S3 shear zones (Fig. 1c). The instrument allows for near-real-time on-site environmental quantification of partial pressures and hence the concentrations of gases dissolved in water^24^. The fluid was collected from the whole area of the seeping shear zone. The fracture was sealed with a plastic foil and waterproof mortar, forcing the fluid to drain by gravity to the GE-MIMS. The sealing was designed to avoid any secondary gas exchange with the atmospheric gases. Background monitoring with GE-MIMS revealed anoxic conditions, confirming that the sampling system was airtight. Then, the collected water was transferred to GE-MIMS membrane contactors (two parallel Micro Modules 3 M Liqui-Cel MM-0.5–1×1 Series), which are specifically designed for low flow rates (from 5–1000 ml/min). Water temperature and total gas pressure in the membranes’ gas head space are determined by the respective sensors. The small head space further maintains a high temporal resolution at the respective water flow rate. He, Ne (in dry gas), Ar, Kr, N_2_, and O_2_ were analyzed every 8 min with an analytical uncertainty of 1–3%. Gas standards from an air-filled Plastigas bag were analyzed every 40 min to calibrate the sample data. Natural background gas concentrations were measured for two days prior to the start of stimulation activities, from May 12^th^ to May 14^th^ (Fig. 2).

The flow rate of the seeping pore water was measured at the outflow of the membrane contactors using a tipping bucket flow gauge connected to a Siemens micrologger starting on May 17 at 6 am before the initiation of HF2. The monitored seeping fracture provided flow rates ranging from 20 to 160 mL/min. These flow rates are lower than the recommended value for air-equilibrated water to maintain the mass balance of the gases in the membrane contactor at gas/water^24^, implying that the partial pressures of the different gas species in the headspace could be controlled by diffusion-limited gas/water exchange across the membranes themselves. This would lead to incomplete gas/water equilibration, especially during periods of low water flow. To avoid any potential effects arising from incomplete gas/water equilibrium within the membrane contactor, we normalized the noble gas concentrations by the measured concentrations of dissolved N_2_, which is only of atmospheric origin in our case study. With ratios of aqueous diffusion coefficients of 3.34 (He/N_2_) and 1.06 (Ar/N_2_), diffusive fractionation would result in an excessive He/N_2_ ratio compared with Ar/N_2_ if the mass balance of the gases in the membrane contactors would be limited by diffusion across the membrane. However, the reverse was observed in the dataset, which indicates that the observed He/N_2_ and Ar/N_2_ ratios are not fractionated by non-equilibrium effects in the membrane contactors, most likely due to the higher gas concentrations in the fluid compared to air-equilibrated water. Therefore, the observed He/N_2_ and Ar/N_2_ ratios accurately reflect the ratios of dissolved He, Ar and N_2_ in the fluid.

The concentrations of He and Ne, as well as the ^3^He/^4^He ratios of individual water samples obtained in standard copper tubes, were analyzed at the noble gas laboratory at ETHZ according to standard analytical protocols. For technical details, the reader is referred to the work of Beyerle *et al*.^38^.

## Supplementary information

Supplementary material.

## Data Availability

The Grimsel ISC Experiment Description is available at 10.3929/ethz-b-000310581. Data used in this publication are available from the ETH Zürich research collection. Partial pressures and discharge rates measured at the GE-MIMS station can be found at 10.3929/ethz-b-000365199. The seismic dataset, as well as hydraulic data of the Grimsel ISC hydraulic fracturing experiments, are available at 10.3929/ethz-b-000276170 and the hydromechanical dataset is available at 10.3929/ethz-b-000328270.
