# Supplementary material for: In situ observation of helium and argon release during fluid-pressure-triggered rock deformation
Source: Sci Rep. 2020 Apr 24;10:6949. doi: 10.1038/s41598-020-63458-x (PMC7181768; doi:10.1038/s41598-020-63458-x)
Supplement: Supplementary file 1 — Supplementary material. [file 41598_2020_63458_MOESM1_ESM.docx]

# Supplementary Material for “In situ observation of helium and argon release during fluid-pressure-triggered rock deformation” by Clément Roques et al.


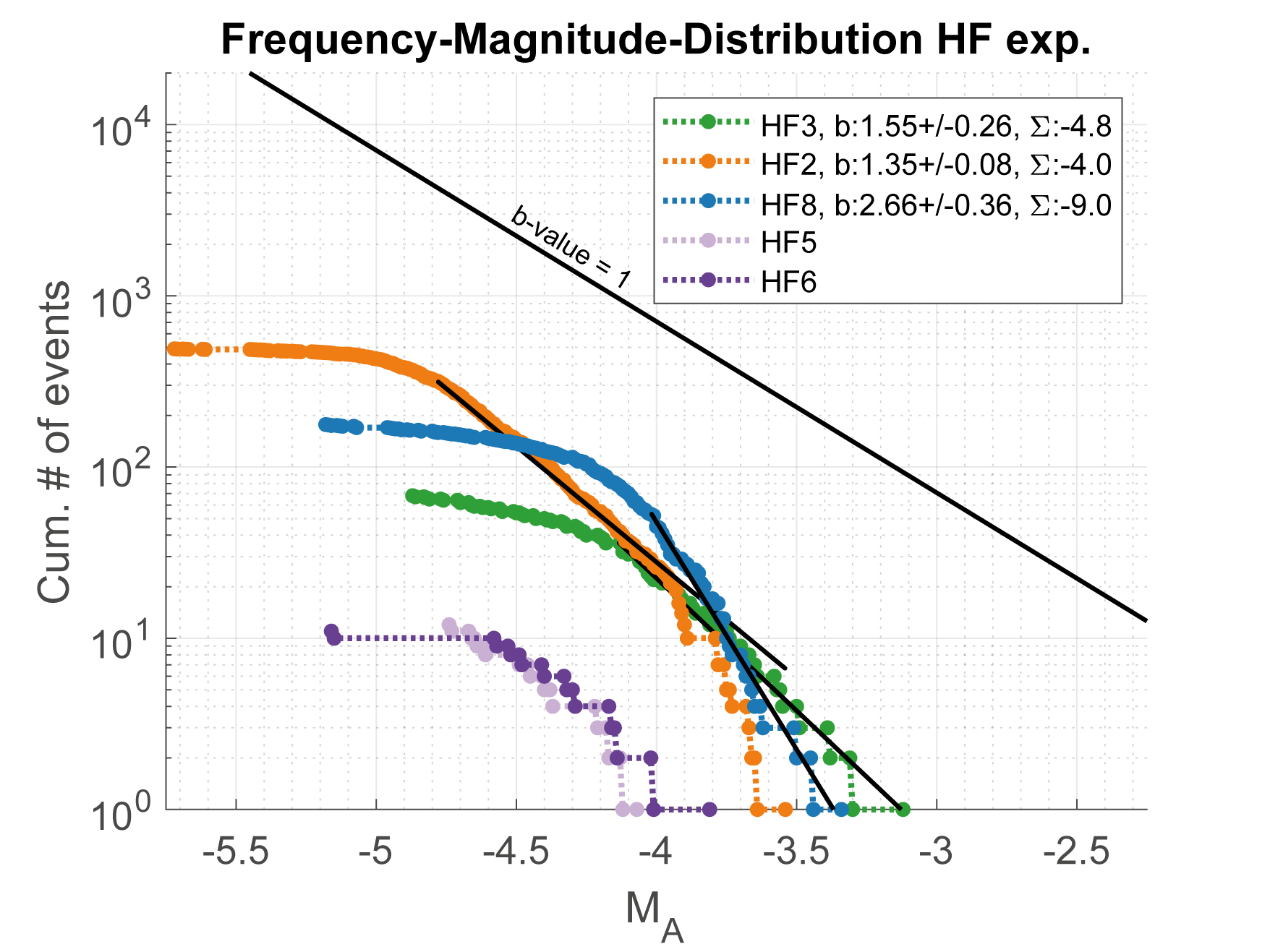


*Figure 1SM: Frequency magnitude distributions for the HF injection experiments along with estimated b-values and seismogenic indices* $\boldsymbol{\Sigma}$*. The injection experiments in the legend are ordered in a chronological manner (adapted from Villiger et al 2019*^1^*).*

Table 1SM: Key metrics and results for each stimulation experiment

|  |  | **HF1** | **HF3** | **HF2** | **HF5** | **HF6** | **HF8** |
| --- | --- | --- | --- | --- | --- | --- | --- |
| **Date** | | 16/05/2017 08:45 | 16/05/2017 13:45 | 17/05/2017 08:20 | 17/05/2017 12:08 | 18/05/2017 08:00 | 18/05/2017 13:00 |
| **Injection**  **location** | **Easting [m]** | 40.2037 | 53.1852 | 42.9028 | 56.9126 | 53.6945 | 61.5029 |
|  | **Northing [m]** | 109.9077 | 99.1799 | 107.6772 | 96.0997 | 115.1335 | 100.2733 |
|  | **Elevation [m]** | 10.4518 | 21.6068 | 12.7711 | 24.8097 | 6.0302 | 22.0441 |
|  | **Distance to outflow at AU Tunnel [m]** | 39.3 | 24.0 | 35.7 | 21.3 | 33.6 | 17.4 |
|  |  |  |  |  |  |  |  |
| **Injection observations** | **Injected Fluid volume [m^3^]** | 1.564 | 0.909 | 0.962 | 0.874 | 1.220 | 1.148 |
|  | **Breakdown pressure [MPa]** | 14.9 | 16.3 | 13.95 | 20.5 | (7.0) | 21.2 |
|  | **Instantaneous shut-in pressure [MPa]** | 4.8 | 5.7 | 4.65 | 5.9 | 5.2 | 4.7 |
|  |  |  |  |  |  |  |  |
| **Monitoring** | **Maximum strain signal [****µε] in S1 shear zone** | -92.4 | 9.8 | -32.7 | 16.9 | 28.5 | -2.5 |
|  | **Maximum strain signal [µε] in S3 Shear zone** | -6.7 | -386.3 | -3.2 | -11.3 | -156.5 | -133.1 |
|  | **Maximum monitored pressure [MPa]** | 1.82 | 2.92 | 3.51 | 2.95 | 2.62 | 1.02 |
|  | **Number detected seismic events** | N/A | 1997 | 2204 | 1969 | 934 | 1501 |
|  | **Number located seismic events (with location accuracy of max. 1.5m)** | N/A | 69 | 497 | 11 | 15 | 182 |
|  | **Average magnitude** | N/A | -4.66 | -4.42 | -4.36 | -4.25 | -4.15 |
|  | **Maximum magnitude** | N/A | -3.54 | -4.07 | -3.81 | -3.34 | -3.12 |
|  | **Activated area [m^2^]** | N/A | N/A | 94.6 | 8 | N/A | 235.7 |
|  | **b-value** | N/A | 1.55 (±0.26) | 1.35 (±0.08) | N/A | N/A | 2.66 (±0.36) |
|  | **Seismogenic index** | N/A | -4.8 | -4.0 | N/A | N/A | -9.0 |

Table 2SM: Concentrations of dissolved gases measured from samples collected in 1982 (from Frick et al., 1992^2,3^) and during this study.

|  | Frick et al., (1992) | | |  | This study - background | | | | | |
| --- | --- | --- | --- | --- | --- | --- | --- | --- | --- | --- |
|  | equilibrium | measured | Σ (meas.) |  | equilibrium | Σ (eq.) | measured | Σ (meas.) | sampling time | analytical method |
| N2 | 1.50E-02 | 1.70E-02 | 1.00E-03 |  | 1.11E-02 | 1.59E-05 | 1.37E-02 | 5.95E-05 | 12-14.05.2017 | in situ GE-MIMS |
| He | 4.00E-08 | 9.30E-07 | - |  | 3.71E-08 | 1.08E-11 | 3.39E-07 | 8.28E-09 | 12-14.05.2017 | in situ GE-MIMS |
| Ne | 1.80E-07 | 8.60E-07 | - |  | - | - | - | - |  |  |
| Ar | 3.50E-07 | 5.00E-04 | 1.00E-05 |  | 2.92E-04 | 4.52E-07 | 3.73E-04 | 4.10E-06 | 12-14.05.2017 | in situ GE-MIMS |
| Kr | 8.00E-08 | 1.20E-07 | - |  | 6.82E-08 | 1.31E-10 | 6.05E-08 | 1.62E-09 | 12-14.05.2017 | in situ GE-MIMS |
| Xe | 1.10E-07 | 1.20E-07 | - |  | - | - | - | - |  |  |
| O2 | - | - | - |  | 5.25E-03 | 8.22E-06 | 9.76E-05 | 8.51E-06 | 12-14.05.2017 | in situ GE-MIMS |
| CH4 | - | - | - |  | - | - | 1.17E-07 | 5.34E-09 | 12-14.05.2017 | in situ GE-MIMS |
| CO2 | - | - | - |  | - | - | 4.70E-05 | 9.89E-06 | 12-14.05.2017 | in situ GE-MIMS |
| 3He/4He | - | - | - |  | - | - | 2.21E-07 | 3.04E-09 | 12.05.2017 | lab mass spectrometer |
| He | - | - | - |  | - | - | 3.94E-07 | 2.50E-09 | 12.05.2017 | lab mass spectrometer |
| Ne | - | - |  |  | - | - | 1.77E-07 | 1.46E-09 | 12.05.2017 | lab mass spectrometer |

# References

1. Villiger, L. *et al.* Influence of reservoir geology on seismic response during decameter scale hydraulic stimulations in crystalline rock. *Solid Earth Discuss.* **2019**, 1–46 (2019).

2. Frick, U. *et al.* *Grimsel Test Site - The Radionuclide Migration Experiment - Overview of Investigations 1985 -1990. PSI Bericht Nr. 120 (Nagra NTB 91-04)*. (1992).

3. Baertschi, P., Antonsen, O., Meier, W. R. & Keil, R. *Untersuchungen an Wasserproben der Sondierbohrunoen SB1-SB4 im Hauptstollen des Pumpkraftwerks Grimsel/Oberaar (Nagra Auftrag), EIR Internal Technical Report TM-44-82-13*. (1982).
